# Older Caregivers’ Positive and Negative Experiences With Caregiving

**DOI:** 10.1093/geroni/igaa057.243

**Published:** 2020-12-16

**Authors:** Latarsha Chisholm, Lynn Unruh, Jacqueline Lamanna

**Affiliations:** 1 University of Central Florida, Orlando, Florida, United States; 2 university of central florida, Orlando, Florida, United States; 3 UCF, Orlando, Florida, United States

## Abstract

U.S. population growth is greatest amongst the oldest-old who are most likely to need temporary or permanent reliance on formal or informal care-giving. With co-survival of older spouses, and greater longevity, informal care-giving is increasingly provided by older adults for other older adults. Little is known about the positive and negative care-giving experiences of older informal caregivers. This observational study examines the relationship between the care-giving situation and the positive and negative perceptions of care-giving in caregivers age 65 and older. A survey of 108 older informal caregivers utilizes well-validated instruments to measure perceptions of two positive (positive self image, positive life) and two negative (stress, uncertainty) experiences due to care-giving. Regressions statistically assess the relationship between care-giving situations and these positive and negative perceptions. We find that while some care-giving situations, such as higher amounts of unpaid support, are related to positive care-giving experiences, others, such as care-giving a recipient with a greater number of ADL deficits, having more care-giving responsibilities, and having increased hours of work due to care-giving, are related to more negative experiences. Some situations, such as lower income and greater involvement in care-giving, are related to both positive and negative perceptions. Higher levels of emotional support were related to mixed positive and less negative perceptions of care-giving. Result indicate that healthcare system and community support is needed to provide opportunities for unpaid support and assistance with caregivers of recipients with a high number of ADL deficits and caregivers who have more care-giving responsibilities.

